# Stability Modelling of mRNA Vaccine Quality Based on Temperature Monitoring throughout the Distribution Chain

**DOI:** 10.3390/pharmaceutics14020430

**Published:** 2022-02-17

**Authors:** Zoltán Kis

**Affiliations:** 1 Department of Chemical and Biological Engineering, The University of Sheffield, Mappin St., Sheffield S1 3JD, UK; z.kis@sheffield.ac.uk; 2 The Sargent Centre for Process Systems Engineering, Department of Chemical Engineering, Imperial College London, South Kensington Campus, London SW7 2AZ, UK

**Keywords:** mRNA vaccines, LNP-mRNA, COVID-19, stability modelling, quality by design, stability related CQAs, supply chain

## Abstract

The vaccine distribution chains in several low- and middle-income countries are not adequate to facilitate the rapid delivery of high volumes of thermosensitive COVID-19 mRNA vaccines at the required low and ultra-low temperatures. COVID-19 mRNA vaccines are currently distributed along with temperature monitoring devices to track and identify deviations from predefined conditions throughout the distribution chain. These temperature readings can feed into computational models to quantify mRNA vaccine critical quality attributes (CQAs) and the remaining vaccine shelf life more accurately. Here, a kinetic modelling approach is proposed to quantify the stability-related CQAs and the remaining shelf life of mRNA vaccines. The CQA and shelf-life values can be computed based on the conditions under which the vaccines have been distributed from the manufacturing facilities via the distribution network to the vaccination centres. This approach helps to quantify the degree to which temperature excursions impact vaccine quality and can also reduce vaccine wastage. In addition, vaccine stock management can be improved due to the information obtained on the remaining shelf life of mRNA vaccines. This model-based quantification of mRNA vaccine quality and remaining shelf life can improve the deployment of COVID-19 mRNA vaccines to low- and middle-income countries.

## 1. Introduction

The detrimental impact of pandemics, such as the COVID-19 pandemic, can be reduced by rapidly mass-vaccinating the population against the pandemic pathogen. The successful COVID-19 mRNA vaccines were developed based on the persistent groundwork laid by devoted scientists such as Dr. Katalin Karikó and many more. However, as of October 2021, COVID-19 vaccines have been administered predominantly in high- and middle-income countries, while low-income countries are left behind [1]. This difference between countries of varying income level is even more pronounced with regards to the use of mRNA vaccines [1]. The deployment of current thermolabile mRNA COVID-19 vaccines in low-income countries is hindered by the high mRNA COVID-19 vaccine selling prices. In addition, distribution challenges can also be expected due to the lack of adequate cold chain infrastructure in low-income countries.

These thermolabile COVID-19 mRNA vaccines require distribution and storage under cold and ultra-cold conditions. However, these cold and ultra-cold chains are prone to faults and failure [2,3,4,5]. Cold chain faults and failures are even more frequent and severe in low- and middle-income countries (LMICs) [5,6,7]. Moreover, vaccine cold chain equipment in LMICs is frequently exposed to harsh environmental conditions, such as extreme temperatures, high humidity and dust, in addition to occasional substandard installation, intermittent power supply, insufficient maintenance capacity and inadequate supplies of replacement/maintenance parts [5,7]. In fact, according to a joint statement from the World Health Organization (WHO) and the United Nations Children’s Fund (UNICEF) in 55 LMICs in 2014, 20% of health facilities did not have cold chain equipment, 14% had non-functional cold chain equipment, 41% had poorly performing equipment, 23% had outdated cold chain technologies, while only 2% had a functional cold chain with optimal technology [5,6]. Besides lacking the adequate infrastructure and physical equipment, cold chain failures in LMICs can also be attributed to: (1) information gaps and the lack of ability to manage flawed information, (2) inadequate training and low knowledge on cold chain management, (3) underfunding and understaffing, (4) lack of vigilance, and (5) failures in decision making, coordination and planning [3,8,9,10,11]. Taken together, these issues have led to the wastage of up to 50% of the vaccines annually [12,13,14]. It is possible that the situation has slightly improved in the past few years, however cold chain issues are likely to cause problems and delays when sending these thermolabile mRNA COVID-19 vaccines to LMICs. 

Given these strict COVID-19 mRNA vaccine cold chain requirements (see Section 2 below), temperature monitoring devices are included in each vaccine shipment, such as the TagAlert Temperature Monitors which accompany Moderna’s COVID-19 mRNA vaccine [15,16,17] and the GPS-enabled thermal sensors that monitor Pfizer’s COVID-19 mRNA vaccine [18,19,20]. These devices track the temperature of the vaccines and indicate whether the temperature of the vaccines during distribution was maintained within the range specified by the vaccine manufacturer. However, these monitoring devices do not provide information about the remaining shelf-life of the vaccine in function of the temperature exposure profiles. Neither do these monitoring devices assess the status of the vaccine critical quality attributes (CQAs) which can be affected during mRNA vaccine distribution. However, the temperature reading from these monitoring devices can feed into computational degradation kinetic models. Therefore, modelling of mRNA degradation kinetics can be feasible [21,22,23,24,25] and with further investigation the impact of temperature exposure profiles on CQAs can be assessed [26,27,28]. Therefore, here solutions are conceptualised to quantify the impact of the distribution conditions, including temperature excursions, on the remaining shelf life and on the stability-related CQAs of these thermolabile mRNA vaccines. The computed values of these stability-related CQAs can be used to determine the overall stability and remaining shelf-life of mRNA vaccines. The aim of this perspective study is to propose a new approach for quantifying mRNA stability-related CQAs and remaining shelf-life based on combining computer models and existing temperature monitoring techniques.

## 2. mRNA Vaccine Formulations and Storage Requirements

The active ingredient or drug substance of Moderna’s and BioNTech/Pfizer’s COVID-19 vaccine is the mRNA which encodes the prefusion stabilized full-length spike glycoprotein of the Wuhan-Hu-1 isolate of SARS-CoV-2 [25,29,30]. This mRNA is single-stranded, 5’-capped and codon optimised. Importantly, the uridine nucleosides are replaced by N1-methylpseudouridine nucleosides [25,29,30]. N1-methylpseudouridine is used because Dr. Katalin Karikó and Drew Weissman has demonstrated that it reduces the level of the innate immune response and at the same time increases protein translation levels [31,32,33]. The Moderna mRNA-1273 vaccine contains 100 µg of mRNA per dose, while the BioNTech/Pfizer BNT162b2 vaccine contains 30 µg of mRNA per dose.

These mRNA molecules are encapsulated into lipid nanoparticles (LNPs) which are placed in an aqueous cryoprotectant buffer [25,29,30]. The composition of these two mRNA vaccine formulations is shown below in Table 1. The ionisable lipids SM-102 and ALC-0315, together with the PEGylated lipids ALC-0159 and PEG2000-DMG are the novel excipients. Since the BioNTech/Pfizer COVID-19 mRNA vaccine obtained emergency use authorisation, its formulation buffer has been updated from the old phosphate buffered saline (PBS) formulation to the new Tris buffer formulation [34]. The new Tris buffer formulation does not contain sodium chloride and potassium chloride, while maintaining the same target pH of 7.4 [34]. Additionally, this new Tris buffer formulation of the BioNTech/Pfizer vaccine comes in two formats to be used with or without dilution for administration at the vaccination centres [35,36]. The vaccine solutions are filled into borosilicate or aluminosilicate glass multidose vials with bromobutyl or chlorobutyl rubber stoppers and aluminium seals. The new BioNTech/Pfizer mRNA vaccine that does not require dilution contains 2.25 mL solution intended for 6 doses, with 0.3 mL per dose. On the other hand, the Moderna mRNA vaccine contains 6.3 mL for 10 doses, with 0.5 mL per dose. These vials are then placed into secondary and tertiary packaging for distribution and low or ultra-low temperatures.

In order to facilitate distribution of its COVID-19 mRNA vaccine, Pfizer has designed special thermal shipping containers that utilise dry ice [18,20,37]. This original PBS formulation of this vaccine requires ultra-cold temperatures of between −90 °C and −60 °C, commonly −80 °C for shipment and longer term storage for up to 6 months, cf. Table 2 [18,20,37,38]. Alternatively, the PBS formulated BioNTech/Pfizer COVID-19 mRNA vaccine can also be transported between −25 °C and −15 °C, commonly −20 °C, and the unpunctured vials can be stored at this temperature for up to 2 weeks [18,20,37,38]. Transportation of this vaccine between 2 °C and 8 °C is also possible, however this should be completed within 12 h [18,20,37,38]. Unpunctured PBS formulated BioNTech/Pfizer COVID-19 mRNA vaccine vials can be kept at 2 °C and 8 °C for up to 1 month, however once punctured and mixed with the diluent these vials need to be used within 6 h at room temperature (8 °C to 25 °C) [18,20,37,38]. Once the vaccine is thawed it should not be frozen again and exposure to sunlight should be avoided [18,20,37,38]. The updated Tris formulation of the BioNTech/Pfizer COVID-19 mRNA vaccine has an enhanced stability profile and can be stored for 9 months at −90 °C to −60 °C, commonly at −80 °C. In addition, this updated formulation can be stored for up to 10 weeks at temperatures between 2 °C and 8 °C, commonly at 4 °C. Punctured vials containing the Tris formulation of the BioNTech/Pfizer mRNA vaccine can be kept at temperatures between 2 °C and 30 °C for 12 hours, thus doubling the time available for administration compared to the original PBS formulation.

On the other hand, Moderna’s COVID-19 mRNA vaccine does not require ultra-cold temperatures for long term storage and transportation. This vaccines is distributed and stored frozen for 6 months at temperatures between −50 °C and −15 °C, commonly at −20 °C [4,15,17,39]. Unpunctured vials may be stored in the refrigerator between 2 °C to 8 °C for up to 30 days and between 8 °C to 25 °C for a total of 24 h [4,15,17,39]. Punctured vials may be stored between 2 °C and 25 °C for up to 12 h [15,17,39]. Moderna’s COVID-19 mRNA vaccine vials cannot be frozen again once thawed, should not be placed on dry ice and prior puncturing vials should not be exposed to sunlight [15,17,39]. 

## 3. mRNA Vaccine Instability and Stability Modelling

mRNA molecules at neutral or slightly alkaline pH, such as in current mRNA vaccine formulations, degrade predominantly via the cleavage of the RNA phosphodiester bonds of the RNA backbone. This 3’, 5’ phosphodiester bond breaks via a transesterification reaction due to the close proximity of the adjacent 2′-hydroxyl group of the ribose moiety to the phosphorus center [21,26,40]. This transesterification reaction occurs via an S_N_2 nucleophilic substitution reaction mechanism, whereby the 2′ oxygen attacks the adjacent phosphorus center. Under alkaline conditions, base catalysis occurs, whereby the 2′-hydroxyl group of the ribose moiety is deprotonated by hydroxide to generate the more nucleophilic 2′-oxyanion group [21,26,40]. As a result of this transesterification reaction, a new bond between the 2′ oxygen and phosphorus is created and the bond between the same phosphorus and 5′ oxygen of the adjacent ribose is cleaved. Thus, the RNA backbone is cleaved and two new ends of the RNA polymer are created; one end has a cyclic 2′,3′- cyclic phosphate while the other end has a 5‘ alkoxide [21,26,40]. This transesterification reaction is also referred to as base-catalyzed hydrolysis or auto-hydrolysis. 

Kinetic models describing mRNA degradation have been developed based on first-order kinetics at physiological pH ranges [21,22,23]. It was also shown that this mRNA degradation reaction follows the Arrhenius behavior [21,24]. Recently, Moderna also stated that the mRNA from their COVID-19 vaccine demonstrated Arrhenius behavior, with first order kinetics [25]. Moreover, the stability profiles from this Moderna mRNA vaccines were shown to be predictable and amenable to modelling [25].

Besides pH, higher order RNA structures (e.g., secondary structures, tertiary structures) can also contribute to the rate of the transesterification reaction [21,22,26,40]. Computational molecular modelling and molecular dynamics simulations are being used to predict RNA structure. These include various atomistic force field methods, broad spectrum of enhanced sampling methods, and coarse-grained modeling [22,26,41,42,43,44,45,46,47,48,49,50]. Single stranded RNA is more prone to hydrolysis than double stranded RNA, however RNAse A enzymes catalyze the cleavage of RNA molecules, including double-stranded RNA, using acid-base hydrolysis [26]. Thus, it is crucial to prevent the contact or interaction of RNAse A with the mRNA. 

The encapsulation of mRNA into LNPs protects the mRNA of current COVID-19 vaccines from the action of RNAse A enzymes when the vaccine is injected into humans, and the LNPs also help the delivery of the RNA into the cells [25,29]. Therefore, the colloidal stability of the mRNA-LNP complexes is also crucial for the quality, safety and efficacy of mRNA vaccines. The LNPs are subject to both chemical and physical instability [27,28,51]. Chemical instability can be caused by oxidation, and temperature- and pH-dependent hydrolysis of the lipids [27,28,51]. Physical instability can occur in the following forms: aggregation, fusion, and leakage of the encapsulated RNA [27,28,51]. However, additional investigation is needed to fully understand the mechanisms of LNP instability [27,52]. Such a detailed mechanistic understanding of LNP instability or the availability of abundant data can support the development of mechanistic or data-driven models, respectively.

## 4. Quality by Design and mRNA Vaccine Stability

Quality by Design (QbD) offers a patient centric approach for consistently delivering high quality, safe and effective vaccines based on product and process understanding [23,53,54]. The critical quality attributes (CQAs) of mRNA vaccines can change over time and under the influence of factors such as temperature, light and shear stress. When mRNA molecules undergo degradation the following CQAs can be affected: RNA sequence integrity, double-stranded RNA (dsRNA) content, poly(A) tail length, poly(A) tail level, 5′ capped RNA percentage and truncated RNA content. In addition, the mRNA-LNP complexes can also be impacted by the above-mentioned factors and the following mRNA-LNP CQAs can undergo changes: RNA encapsulation, LNP size, LNP polydispersity, and lipid-RNA adduct impurities. Consequently, alterations of these CQAs can impact additional CQAs, such as immunogenicity and potency, ultimately leading to changes in product quality, safety and efficacy. The stability of mRNA vaccines can be quantified based on quantifying these individual stability related CQAs. Acceptance criteria or thresholds can be defined for each CQA, and the mRNA vaccine can be considered stable and within the shelf-life only if the acceptance criteria for each individual CQA is met.

The assessment and measurement of stability-related CQAs requires specialised assays and equipment, as shown below in Table 3. Therefore, the quantification of stability-related CQAs cannot be done routinely at the use-point of the vaccine following vaccine distribution. To prevent mRNA degradation and alterations in the mRNA CQAs and mRNA-LNP CQAs, these vaccines are handled and distributed under well-controlled conditions (see Section 2). This is meant to reduce the impact of factors such as temperature, time, light and shear stress on the stability-related mRNA CQAs and mRNA-LNP CQAs. However, vaccine distribution supply chains in low-income countries can be unreliable as described in the Introduction section and consequently these CQA-impacting factors can be less well-controlled. This can lead to deterioration in vaccine quality and loss of vaccine doses.

## 5. Supply Chain of mRNA Vaccines

Current mRNA vaccines are thermolabile and their quality can deteriorate during both manufacturing and distribution [25,59,71]. Vaccines, including mRNA vaccines, are manufactured in two main stages, and following this, vaccines are distributed via a network of storage and transportation steps to the vaccination centres [59,71,72,73]. The two stages of vaccine manufacturing are the drug substance manufacturing (primary manufacturing) and drug product manufacturing (secondary manufacturing, fill-to-finish) [59,71,72]. These two manufacturing stages generally take place at different locations, often in different countries, thus the vaccine drug substance needs to be stored and transported between these two locations. Following fill-to-finish, the packaged vaccines are stored at the secondary manufacturing facility and then are transported to the central/national stores. These central/national stores can be in the country of secondary manufacturing, but more commonly vaccines are shipped to a different country. If vaccines are shipped to a different country or continent, airplanes are used for long-distance transportation in combination with road transportation at both ends, from the secondary manufacturing facility to the airport and from the airport to the central/national store. From the national stores, the vaccines are transported usually to several provincial/regional stores, from there to tens to hundreds of district stores and then to generally thousands of hospitals and vaccination centres [73,74,75,76,77,78,79,80]. This is a 4-tier distribution network, however 3-tier and 5-tier vaccine distribution networks also exist, but these tend to be less common [79,80].

## 6. A Model-Based Quantification of the mRNA Vaccine Shelf Life and Stability-Related CQAs

In principle, it is possible to establish mathematical relationships between the CQAs of mRNA vaccines and the factors or parameters that lead to their alteration during the distribution chain. For example, kinetic equations can be built to describe degradation reactions in function of time and temperature [21,22,23,24,25,81]. For this, the following simple kinetic equation can be used to describe the rate of the degradation reaction:

(1) $$r_{deg}=k\;{\left[mRNA\right]}^{m}$$

where:

“*r_deg_*” is the reaction rate for the degradation reaction, units depend on the order of reaction

“*k*” is the rate constant, its units depend on the order of reaction

“[*mRNA*]” is the concentration of the active, functional process intermediates

“*m*” is the order of the reaction.

The order of the reaction can be determined experimentally by measuring the reaction rate at two different starting mRNA concentrations at a constant temperature [82,83]. This experiment can be carried out at multiple temperatures to confirm that the order of reaction and reaction mechanism remains the same when measuring the rate at different temperatures. The obtained order of reaction can then be checked by carrying out a degradation experiment at a constant temperature and measuring the concentration of the mRNA over time. The shape of the concentration curve in function of time can confirm the order of the reaction [82,83]. Next, the rate constant *k* can be determined from the degradation rate law.

To relate the reaction rate to temperature, the Arrhenius equation is commonly used [84,85,86,87,88,89]:

(2) $$k=A\;e^{\frac{-E_{a}}{RT}}$$

where:

“*k*” is the rate constant, units depend on the order of reaction

“*A*” is the pre-exponential factor, a constant for each chemical reaction. According to collision theory, *A* is the frequency of collisions in the correct orientation. *A* has the same unit as *k*;

“*T*” is the absolute temperature, in Kelvin;

“*R*” is the universal gas constant, can be expressed as 8.3144 J⋅K^−1^⋅mol^−1^;

“*Ea*” is the activation energy for the reaction, expressed in the same units as *RT*;

To check whether the reaction follows the Arrhenius equation, the Arrhenius equation can be linearised by taking its natural logarithm. This gives the following equation:

(3) $$\ln k=-\frac{E_{a}}{R}\frac{1}{T}+\ln A$$

If the plot of lnk in function of $\frac{1}{T}$ gives a line, that means that the reaction has an Arrhenius behaviour. The reaction rate constant *k* and the temperature *T* can be determined experimentally [82,83]. By plotting lnk in function of $\frac{1}{T}$, $\frac{E_{a}}{R}$ will be represented by the slope (gradient) of the line, whereas lnA will be the intercept. Thus, by knowing all the components of the equation, including the universal gas constant *R*, the equation can now be solved and the activation energy *E_a_* as well as the pre-exponential factor *A* can be determined. 

Such stability modelling approaches have been already used in the past, predominantly for protein-based biopharmaceuticals and conventional (non mRNA) vaccines [81,84,90]. It was also demonstrated that the mRNA degradation reaction follows the Arrhenius behavior [21,24]. Furthermore, Moderna has stated that mRNA degradation rate demonstrates Arrhenius behaviour with first order kinetics [25]. According to Moderna, the mRNA stability profiles were demonstrated to be predictable and amenable to modelling [25]. 

If new data will show that the first-order kinetics and the Arrhenius equation will not describe the degradation of mRNA vaccines accurately, then alterations to these models can be made, for example by changing the order of the reaction or using a modified version of the Arrhenius equation. The model structure should be developed by investigating the underlying mechanisms and the kinetics. The possibility of multiple parallel or stepwise reactions should also be considered. The impact of phase transitions (e.g., solid to liquid) on the mRNA vaccine stability related CQAs should also be evaluated.

The correct model can in principle quantify the extent of mRNA degradation under any combination of time and temperature. 

Following their conception, determination of the appropriate model structure and order of reaction, models need to be calibrated and then validated to make sure that they are realistic. Model calibration, or model fitting, implies adjusting the model parameters to the experimental data to optimise the fit between the experimental data, and the model and to maximise the predictive power of the model. Model validation entails the use of additional experimental data to check if the model has the desired predictive power. Such a model needs to be validated under real-life conditions. For model calibration and validation, two experimental approaches can be used: accelerated stability studies under isothermal conditions and temperature ramping experiments [81,90].

During accelerated degradation studies under isothermal conditions the degradation reaction is observed at high temperatures. This increases the reaction rate and reduces the experimentation time. For such studies, the mRNA is incubated at high temperatures and the change in the concentration of the mRNA is measured over time. Thus, the reaction rate constant *k* can be determined from Equation (1). By knowing the temperature, the line from Equation (3) can be plotted and from this 2D plot the activation energy *E_a_* as well as the pre-exponential factor *A* can be determined. Therefore, by knowing the degradation rate at various high temperatures and after confirming that the temperature-rate dependency remains valid at different temperatures, the relationship can be extrapolated to determine the degradation rate at low temperatures. 

On the other hand, during temperature ramping experiments, the temperature is increased either step-wise or continuously at a given heating rate over a define temperature range. During this experiment, changes in the biophysical properties of the mRNA can be assessed by monitoring the degradation extent and/or by monitoring the enthalpy and free energy or another thermodynamic parameter [84,91,92,93]. Temperature ramping experiments can be carried out within a few hours and this is an advantage compared to accelerated stability studies under isothermal conditions. Therefore, temperature ramping experiments are commonly used for biopharmaceutical formulation development and for screening of stabilising conditions [84,91,92,93]. The models should also be validated using real-world supply chain conditions. 

**Figure 1:**
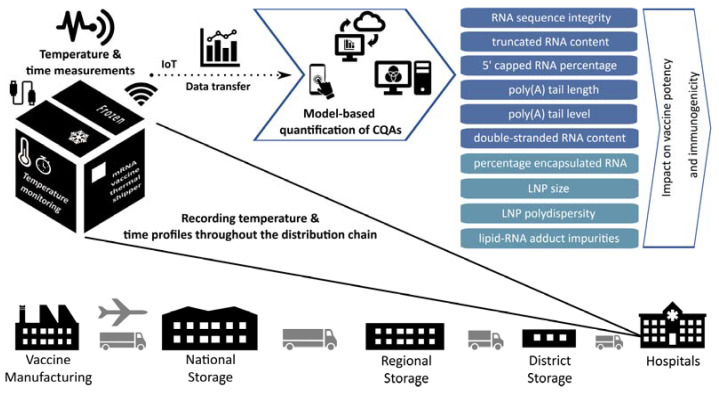
Illustration of the proposed model-based quantification of mRNA vaccine stability related CQAs and remaining shelf life. The temperature of COVID-19 mRNA vaccines is currently monitored throughout the distribution chain. Models can be developed, calibrated, validated and implemented to compute the values of stability related CQAs based on temperature and time measurements. These models can be cloud based and they could receive the data via the internet. After the results have been computed these can be returned to the user’s computer or mobile device (e.g., smartphone). These results will contain the quantitative values for each CQA and also the remaining shelf life.

Once such a model will be calibrated and successfully validated, this model will be ready to be applied to compute the stability-related CQAs of the mRNA vaccine at any possible combinations of temperature and time duration during vaccine distribution, cf. Figure 1. For this, the model will take in measurements from temperature monitoring devices which are already included in each vaccine shipment, i.e., the TagAlert Temperature Monitors which accompany Moderna’s COVID-19 mRNA vaccine [15,16,17] and the GPS-enabled thermal sensors that monitor Pfizer’s COVID-19 mRNA vaccine [18,19,20]. The models can also be cloud-based and the information from the temperature monitoring devices can be transferred to the models via the internet. Based on these measurements, the calibrated and validated models can compute values of the CQAs, such as those shown in Table 3. The model-computed CQA values can be returned via the internet to the computer or mobile device (e.g., smartphone) of the user or vaccinator. A QbD design space can also be established to illustrate the impact of storage and transportation parameters, such as temperature and time, on the individual stability-related CQAs [23,81]. This design space will illustrate the combination of time and temperature that the mRNA vaccine can be exposed to in the future. 

These models can also compute the remaining shelf life of vaccines by quantifying how high above the allowable threshold the CQA values are and therefore are able to predict when these CQAs would go outside of the acceptable limits. This will help to better understand which vaccines are usable even if the cold chain was partially compromised and if temperature excursions occurred. Therefore, this model-based stability quantification can reduce vaccine wastage while still guaranteeing that the vaccines are effective and safe to use. Moreover, by knowing the remaining vaccine shelf-life, the vaccine stocks can also be managed more effectively. These stability QbD models are in principle disease agnostic and can be used to quantify the stability-related CQAs and remaining shelf life of mRNA vaccines against a wide range of diseases beyond COVID-19. If the mRNA vaccine formulation and length of mRNA molecule changes considerably, the models might need to be recalibrated and revalidated or even new model architectures might need to be developed.

## 7. Remaining Challenges and Potential Solutions

As shown above, mRNA degradation can be modelled using kinetic equations and this facilitates the quantification of CQAs such as RNA sequence integrity, truncated RNA content, 5′ capped RNA percentage, and to some extent poly(A) tail length and poly(A) tail level. However, values for other CQAs can be more challenging to quantify using computational models based on currently available mechanistic understanding and available data. These CQAs include, double-stranded RNA content, percentage encapsulated RNA, LNP polydispersity and lipid-RNA adduct impurities. The double-stranded RNA content can in principle be modelled by establishing statistical or data-driven relationships between factors such as temperature-time profiles and double-stranded RNA content based on experimental or real-world data. However, this would require a large amount of data from a wide range of temperature and time conditions. Alternatively, in principle, ab initio molecular modelling could also be used to predict secondary structures and double stranded RNA formation in function of temperature and time profiles [22,26,41,42,43,44,45,46,47,48,49,50,94,95,96]. There is also some degree of sequence flexibility that can be used to control secondary structure formation, due to the codon redundancy, choice of modified nucleotides, and changes that can be implemented into the 5′ UTR and 3′ UTR [97,98].

Stability related CQAs pertaining to the LNP can also be more challenging to quantify using modelling. Thereby, besides statistical and data-driven approaches, models also encompassing chemical and physical degradation pathways can also be evaluated. These models should quantify the oxidation and hydrolysis of lipids, as well as assess LNP aggregation, fusion, leakage of the encapsulated RNA, together with biophysical and morphological changes in the LNPs [27,28,51,52,68,99]. Moreover, models for reactions between lipids and RNA can also be developed to describe the formation of the recently observed lipid-mRNA adducts that can occur in mRNA vaccine formulations [70]. If necessary, models can also be developed to quantify the impact of additional factors, such as light and shear stress, on the stability-related CQAs. The impact of phase transitions (e.g., solid to liquid) can also be experimentally assessed and then potentially modelled. Finally, the presence of autocatalytic events or other deviations from the model-predicted evolution of the stability-related CQAs should also be carefully evaluated as part of the model validation exercise.

Besides the use of models for better quantifying the remaining shelf life and the values for the stability-related CQAs, an alternative and crucial approach is to increase the thermostability of mRNA vaccine formulations. This is indeed a topic of highly active research and it could lead to more thermostable mRNA vaccine formulations in the long term [100,101]. Another solution for enabling large scale vaccination programs in LMICs is to improve the vaccine distribution cold chains in these countries. However, this is extremely challenging to achieve due to lack of adequate road and electricity infrastructure, lack of suitable cold chain equipment, shortages of qualified cold chain operating and maintaining personnel, and lack of funding. There is also the possibility of using the cold chains from other sectors, such as food, fishery and agricultural sectors to distribute COVID-19 mRNA vaccines more effectively [13]. This cross-sectoral use of supply chains can increase the distribution capacity and to some extent the reliability of cold and ultra-cold mRNA vaccine chains. However, incidents, faults and failures can still occur. Taken together these efforts could help with enabling the delivery and usage of vaccines in LMICs, once these COVID-19 mRNA vaccines are made more affordable to LMICs.

## 8. Conclusions

Current COVID-19 mRNA vaccines are thermosensitive and need to be distributed via cold or ultra-cold chains to minimise their degradation and change in quality. However, such cold and ultra-cold chains in LMICs are often not adequate or are unreliable. COVID-19 mRNA vaccines are currently distributed along with temperature monitoring devices to track and identify deviations from predefined conditions throughout the distribution chain. Given the sub-optimal cold chain infrastructure in LMICs, a large proportion of mRNA COVID-19 vaccines could be wasted due to temperature excursions during the distribution chain. It is not well understood how various temperature excursion profiles could impact mRNA vaccine CQAs and the remaining shelf life of the mRNA vaccines. To address this, a model-based quantification of the CQAs and consequently the remaining shelf life is proposed. This can be achievable given that COVID-19 mRNA vaccine shipments already contain temperature monitoring devices and that Moderna has already proposed a model which calculates vaccine degradation in function of time and temperature. In this article, a kinetic model is conceptualised for describing mRNA degradation and an approach is presented for implementing this model into the vaccine distribution chain. These models will take in temperature and time readings from the vaccine shipments and can compute the CQAs and the remaining shelf life of mRNA vaccines. Remaining challenges are also discussed, highlighting potential limitations of such models for quantifying values for certain stability-related CQAs, such as double stranded RNA content and LNP-related CQAs that can be impacted by temperature. Potential modelling approaches are also presented for difficult to quantify CQAs alongside additional improvements that could help the deployment of COVID-19 mRNA vaccines in LMICs. The modelling approaches presented here can help to reduce vaccine wastage, help to better manage vaccines stocks, and more accurately quantify vaccine quality when distributing thermolabile mRNA vaccines to locations where the cold or ultra-cold chain is sub-optimal.

## Figures and Tables

**Table 1:** Composition of the Moderna and BioNTech/Pfizer COVID-19 mRNA vaccine [25,29,30,34,35,36].

Component	Moderna COVID-19 mRNA Vaccine	BioNTech/Pfizer COVID-19 mRNA Vaccine–Original PBS Formulation	BioNTech/Pfizer COVID-19 mRNA Vaccine–Updated Tris Formulation
Active ingredient	nucleoside-modified mRNA-1273 *	nucleoside-modified BNT162b2 mRNA *	nucleoside-modified BNT162b2 mRNA *
Functional, ionisable lipid	SM-102 (heptadecan-9-yl 8-((2-hydroxyethyl)(6-oxo-6-(undecyloxy)hexyl)amino)octanoate)	ALC-0315 (4-hydroxybutyl)azanediyl)bis(hexane-6,1-diyl)bis(2-hexyldecanoate)	ALC-0315 (4-hydroxybutyl)azanediyl)bis(hexane-6,1-diyl)bis(2-hexyldecanoate)
Functional lipid	PEG2000-DMG (1,2-dimyristoyl-rac-glycero-3-methoxypolyethylene glycol-2000)	ALC-0159 (2-[(polyethylene glycol)-2000]-N,N-ditetradecylacetamide)	ALC-0159 (2-[(polyethylene glycol)-2000]-N,N-ditetradecylacetamide)
Structural lipid	DSPC (1,2-distearoyl-sn-glycero-3-phosphocholine)	DSPC (1,2-Distearoyl-sn-glycero-3-phosphocholine)	DSPC (1,2-Distearoyl-sn-glycero-3-phosphocholine)
Structural lipid	Cholesterol	Cholesterol	Cholesterol
Cryoprotectant	Sucrose	Sucrose	Sucrose
Buffer component	Tris (Tromethamine)	Phosphate-Buffered Saline (PBS)	Tris (Tromethamine)
Buffer component (s)	Tris-HCL (tris(hydroxymethyl)aminomethane-hydrochloride), sodium acetate, acetic acid	Disodium phosphate dihydrate, Potassium dihydrogen phosphate, potassium chloride, sodium chloride	Tris-HCL (tris(hydroxymethyl)aminomethane-hydrochloride)
Buffer component	water for injections	water for injections	water for injections
pH	7.5	6.9–7.9	7.4

* These single-stranded mRNA sequences are 5′ capped, codon optimised, and they encode the prefusion stabilized full-length spike glycoprotein of the Wuhan-Hu-1 isolate of SARS-CoV-2.

**Table 2:** Storage and transportation condition comparison for the regulatory-approved COVID-19 mRNA vaccines [4,15,17,18,20,36,37,38,39].

Condition	Moderna COVID-19 mRNA Vaccine	BioNTech/Pfizer COVID-19 mRNA Vaccine–Original PBS Formulation	BioNTech/Pfizer COVID-19 mRNA Vaccine–Updated Tris Formulation
Ultra-cold frozen unopened vials	Not required	−90 °C to −60 °C, commonly −80 °C for six months	−90 °C to −60 °C, commonly −80 °C for nine months
Cold frozen unopened vials	−50 °C to −15 °C, commonly −20 °C for six months	−25 °C to −15 °C, commonly −20 °C, single period of two weeks	−25 °C to −15 °C, commonly −20 °C, single period of two weeks
Thawed unopened vials	2 °C to 8 °C, commonly 4 °C for 30 days *	2 °C to 8 °C, Commonly 4 °C for one month	2 °C to 8 °C, commonly 4 °C for 10 weeks
Thawed punctured vials	2 °C to 25 °C, within 12 h	8 °C to 25 °C, within 6 h	2 °C to 30 °C, within 12 h

* Alternatively, the Moderna COVID-19 mRNA vaccine can also be stored between 8 °C and 25 °C for a total of 24 h.

**Table 3:** List of mRNA-LNP vaccine critical quality attributes related to stability and the analytical methods required to quantify these.

Critical Quality Attribute	Analytical Methods	Ref.
RNA sequence integrity	Capillary Electrophoresis (CE), analytical high-performance liquid chromatography (HPLC), analytical ultra-high-performance liquid chromatography (UHPLC)	[55,56,57,58,59]
truncated RNA content	CE, analytical HPLC, analytical UHPLC	[55,56,57,58,59]
5′ capped RNA percentage	Analytical HPCL, liquid chromatography-mass spectrometry (LC-MS), nuclease digestion followed by tandem mass spectrometry (MS/MS) quantitation	[25,29,56,60,61]
poly(A) tail length	LC-MS, reverse-phase HPLC and mass spectrometry (RP-HPLC-MS), CE	[29,55,62,63]
poly(A) tail level	Analytical HPLC, droplet digital polymerase chain reaction (ddPCR), MS	[29,56,64]
double-stranded RNA content	Immunoblotting, ELISA, RP-HPLC-MS	[27,29,57,65,66]
percentage encapsulated RNA	Absorbance assay, ribogreen assay, ion exchange HPLC, Raman spectroscopy, size-exclusion chromatography with multiangle light scattering (SEC-MALS)	[27,56,67,68]
LNP size	dynamic light scattering (DLS), nanoparticle tracking analysis, SEC-MALS	[27,56,59,67,68,69]
LNP polydispersity	DLS, nanoparticle tracking analysis, SEC-MALS	[27,56,59,67,68,69]
lipid-RNA adduct impurities	Ion pair RP-HPLC, HPLC, UPLC	[25,70]

## References

[1] Duke Global Health Innovation Center COVID-19 Vaccine Manufacturing (2021). The Launch and Scale Speedometer. https://launchandscalefaster.org/covid-19/vaccinemanufacturing.

[2] Rogers B., Dennison K., Adepoju N., Dowd S., Uedoi K. (2010). Vaccine Cold Chain: Part Proper Handling and Storage of Vaccine. AAOHN J..

[3] Lin Q., Zhao Q., Lev B. (2020). Cold chain transportation decision in the vaccine supply chain. Eur. J. Oper. Res..

[4] Grau S., Ferrández O., Martín-García E., Maldonado R. (2021). Accidental Interruption of the Cold Chain for the Preservation of the Moderna COVID-19 Vaccine. Vaccines.

[5] Lennon P., Atuhaire B., Yavari S., Sampath V., Mvundura M., Ramanathan N., Robertson J. (2017). Root cause analysis underscores the importance of understanding, addressing, and communicating cold chain equipment failures to improve equipment performance. Vaccine.

[6] World Health Organization (WHO)—United Nations Children’s Fund (UNICEF) (2016). Achieving Immunization Targets with the Comprehensive Effective vaccine Management (EVM) Framework.

[7] World Health Organization (2014). Immunization Supply Chain and Logistics: A Neglected but Essential System for National Immunization Programmes: A Call-to-Action for National Programmes and the Global Community by the WHO Immunization Practices Advisory Committee.

[8] Comes T., Bergtora Sandvik K., Van de Walle B. (2018). Cold chains, interrupted: The use of technology and information for decisions that keep humanitarian vaccines cool. J. Humanit. Logist. Supply Chain Manag..

[9] Hibbs B.F., Miller E., Shi J., Smith K., Lewis P., Shimabukuro T.T. (2018). Safety of vaccines that have been kept outside of recommended temperatures: Reports to the Vaccine Adverse Event Reporting System (VAERS). Vaccine.

[10] Yassin Z.J., Nega H.Y., Derseh B.T., Yehuala Y.S., Dad A.F. (2019). Knowledge of Health Professionals on Cold Chain Management and Associated Factors in Ezha District, Gurage Zone, Ethiopia. Scientifica.

[11] Bogale H.A., Amhare A.F., Bogale A.A. (2019). Assessment of factors affecting vaccine cold chain management practice in public health institutions in east Gojam zone of Amhara region. BMC Public Health.

[12] World Health Organization (2005). Monitoring Vaccine Wastage at Country Level.

[13] UN Environment Programme Why Optimized Cold-Chains Could Save a Billion COVID Vaccines. https://www.unep.org/news-and-stories/story/why-optimized-cold-chains-could-save-billion-covid-vaccines.

[14] GAVI Cold Supply for Hot Demand: Transforming the Market for Cold Chain Equipment in the World’s Poorest Countries (2017). GAVI, The Vaccine Alliance. https://www.gavi.org/vaccineswork/cold-supply-hot-demand.

[15] (2021). Centers for Disease Control and Prevention Moderna COVID-19 Vaccine—Storage and Handling Summary; Atlanta, GA, USA. https://www.cdc.gov/vaccines/covid-19/info-by-product/moderna/downloads/storage-summary.pdf.

[16] Sensitech Inc (2021). TagAlert Enhanced: The Electronic Alternative for Cost-Effective Temperature Monitoring Down to −30 °C.; Beverly, MA, USA. https://www.sensitech.com/en/media/Indicators_TagAlert_Enhanced_LS_0921_Web_tcm878-140468.pdf.

[17] (2021). Ministry of Health—Ontario Canada COVID-19: Vaccine Storage and Handling Guidance Highlights of Changes; Toronto, ON, Canada. https://www.health.gov.on.ca/en/pro/programs/publichealth/coronavirus/docs/vaccine/vaccine_storage_handling_pfizer_moderna.pdf.

[18] UNICEF, World Health Organization (2021). Training on Handling, Storing and Transporting Pfizer BioNTech COVID-19 Vaccine COMIRNATY® (Tozinameran).

[19] Pfizer Inc Manufacturing and Distributing the COVID-19 Vaccine. https://www.pfizer.com/science/coronavirus/vaccine/manufacturing-and-distribution.

[20] (2021). Centers for Disease Control and Prevention Pfizer-BioNTech COVID-19 Vaccine: Storage and Handling Summary; Atlanta, GA, USA. https://www.cdc.gov/vaccines/covid-19/info-by-product/pfizer/downloads/storage-summary.pdf.

[21] Li Y., Breaker R.R. (1999). Kinetics of RNA Degradation by Specific Base Catalysis of Transesterification Involving the 2′-Hydroxyl Group. J. Am. Chem. Soc..

[22] Wayment-Steele H.K., Kim D.S., Choe C.A., Nicol J.J., Wellington-Oguri R., Watkins A.M., Parra Sperberg R.A., Huang P.-S., Participants E., Das R. (2021). Theoretical basis for stabilizing messenger RNA through secondary structure design. Nucleic Acids Res..

[23] Van de Berg D., Kis Z., Behmer C., Samnuan K., Blakney A., Kontoravdi C., Shattock R., Shah N. (2021). Quality by Design modelling to support rapid RNA vaccine production against emerging infectious diseases. NPJ Vaccines.

[24] Fabre A.-L., Colotte M., Luis A., Tuffet S., Bonnet J. (2014). An efficient method for long-term room temperature storage of RNA. Eur. J. Hum. Genet..

[25] European Medicines Agency (2021). Assessment Report—COVID-19 Vaccine Moderna—Common Name: COVID-19 mRNA Vaccine (Nucleoside-Modified).

[26] Šponer J., Bussi G., Krepl M., Banáš P., Bottaro S., Cunha R.A., Gil-Ley A., Pinamonti G., Poblete S., Jurečka P. (2018). RNA Structural Dynamics As Captured by Molecular Simulations: A Comprehensive Overview. Chem. Rev..

[27] Schoenmaker L., Witzigmann D., Kulkarni J.A., Verbeke R., Kersten G., Jiskoot W., Crommelin D.J.A. (2021). mRNA-lipid nanoparticle COVID-19 vaccines: Structure and stability. Int. J. Pharm..

[28] Fan Y., Marioli M., Zhang K. (2021). Analytical characterization of liposomes and other lipid nanoparticles for drug delivery. J. Pharm. Biomed. Anal..

[29] European Medicines Agency (2021). Assessment report—Comirnaty—Common Name: COVID-19 mRNA Vaccine (Nucleoside-Modified).

[30] World Health Organisation (WHO) (2021). Recommendation for an Emergency Use Listing of COVID-19 mRNA Vaccine (Nucleoside Modified) Submitted by Moderna Biotech (Spain).

[31] Karikó K., Buckstein M., Ni H., Weissman D. (2005). Suppression of RNA Recognition by Toll-like Receptors: The Impact of Nucleoside Modification and the Evolutionary Origin of RNA. Immunity.

[32] Karikó K., Muramatsu H., Welsh F.A., Ludwig J., Kato H., Akira S., Weissman D. (2008). Incorporation of pseudouridine into mRNA yields superior nonimmunogenic vector with increased translational capacity and biological stability. Mol. Ther..

[33] Karikó K., Muramatsu H., Keller J.M., Weissman D. (2012). Increased Erythropoiesis in Mice Injected with Submicrogram Quantities of Pseudouridine-containing mRNA Encoding Erythropoietin. Mol. Ther..

[34] European Medicines Agency (2021). CHMP Assessment Report on Group of an Extension of Marketing Authorisation and Variations—Comirnaty.

[35] Jacqueline A. (2022). O’Shaughnessy Letter to Pfizer Inc. Mr. Amit Patel.

[36] ModernaTX I. (2022). Product Information as Approved by CHMP on 13 January 2022, Pending Endorsement by the European Commission—Annex I—Summary of Product Characteristics. https://www.ema.europa.eu/en/documents/product-information/comirnaty-epar-product-information_en.pdf.

[37] Centers for Disease Control and Prevention (2021). Pfizer-BioNTech COVID-19 Vaccine: Transporting Vaccine for Vaccination Clinics Held at Satellite, Temporary, or Off-Site Locations.

[38] Centers for Disease Control and Prevention (2021). Pfizer-BioNTech COVID-19 Vaccine: Vaccine Preparation and Administration Summary.

[39] Moderna Inc (2021). Moderna COVID-19 Vaccine Storage & Handling.

[40] Oivanen M., Kuusela S., Lönnberg H. (1998). Kinetics and Mechanisms for the Cleavage and Isomerization of the Phosphodiester Bonds of RNA by Brønsted Acids and Bases. Chem. Rev..

[41] Taylor W.R. (2005). Modelling molecular stability in the RNA world. Comput. Biol. Chem..

[42] Lorenz R., Bernhart S.H., Höner zu Siederdissen C., Tafer H., Flamm C., Stadler P.F., Hofacker I.L. (2011). ViennaRNA Package 2. Algorithms Mol. Biol..

[43] Zadeh J.N., Steenberg C.D., Bois J.S., Wolfe B.R., Pierce M.B., Khan A.R., Dirks R.M., Pierce N.A. (2011). NUPACK: Analysis and design of nucleic acid systems. J. Comput. Chem..

[44] Reuter J.S., Mathews D.H. (2010). RNAstructure: Software for RNA secondary structure prediction and analysis. BMC Bioinform..

[45] Do C.B., Woods D.A., Batzoglou S. (2006). CONTRAfold: RNA secondary structure prediction without physics-based models. Bioinformatics.

[46] Terai G., Kamegai S., Asai K. (2016). CDSfold: An algorithm for designing a protein-coding sequence with the most stable secondary structure. Bioinformatics.

[47] Cohen B., Skiena S. (2003). Natural selection and algorithmic design of mRNA. J. Comput. Biol..

[48] Washietl S., Hofacker I.L., Stadler P.F., Kellis M. (2012). RNA folding with soft constraints: Reconciliation of probing data and thermodynamic secondary structure prediction. Nucleic Acids Res..

[49] Zarringhalam K., Meyer M.M., Dotu I., Chuang J.H., Clote P. (2012). Integrating chemical footprinting data into RNA secondary structure prediction. PLoS ONE.

[50] Cordero P., Das R. (2015). Rich RNA Structure Landscapes Revealed by Mutate-and-Map Analysis. PLoS Comput. Biol..

[51] Kim J., Eygeris Y., Gupta M., Sahay G. (2021). Self-assembled mRNA vaccines. Adv. Drug Deliv. Rev..

[52] Crommelin D.J.A., Anchordoquy T.J., Volkin D.B., Jiskoot W., Mastrobattista E. (2021). Addressing the Cold Reality of mRNA Vaccine Stability. J. Pharm. Sci..

[53] Kis Z., Kontoravdi C., Dey A.K., Shattock R., Shah N. (2020). Rapid development and deployment of high-volume vaccines for pandemic response. J. Adv. Manuf. Process..

[54] (2012). CMC-Vaccines Working Group A-Vax: Applying Quality by Design to Vaccines. https://www.dcvmn.org/IMG/pdf/a-vax-applying-qbd-to-vaccines_2012.pdf.

[55] (2021). Agilent Technologies Nucleic acid Analysis for Sample Quality Assessment using the Agilent Fragment Analyzer Systems. https://www.agilent.com/cs/library/applications/application-nucleic-acid-qc-fragment-analyzer-5994-2813en-agilent.pdf.

[56] Poveda C., Biter A.B., Bottazzi M.E., Strych U. (2019). Establishing Preferred Product Characterization for the Evaluation of RNA Vaccine Antigens. Vaccines.

[57] Gagnon P. (2020). Purification of Nucleic Acids—A Handbook for Purification of DNA Plasmids and mRNA for Gene Therapy and Vaccines.

[58] Schroeder A., Mueller O., Stocker S., Salowsky R., Leiber M., Gassmann M., Lightfoot S., Menzel W., Granzow M., Ragg T. (2006). The RIN: An RNA integrity number for assigning integrity values to RNA measurements. BMC Mol. Biol..

[59] (2021). WHO Evaluation of the Quality, Safety and Efficacy of Messenger RNA Vaccines for the Prevention of Infectious Diseases: Regulatory Considerations.

[60] Beverly M., Dell A., Parmar P., Houghton L. (2016). Label-free analysis of mRNA capping efficiency using RNase H probes and LC-MS. Anal. Bioanal. Chem..

[61] Henderson J.M., Ujita A., Hill E., Yousif-Rosales S., Smith C., Ko N., McReynolds T., Cabral C.R., Escamilla-Powers J.R., Houston M.E. (2021). Cap 1 Messenger RNA Synthesis with Co-transcriptional CleanCap(^®^) Analog by In Vitro Transcription. Curr. Protoc..

[62] Beverly M., Hagen C., Slack O. (2018). Poly A tail length analysis of in vitro transcribed mRNA by LC-MS. Anal. Bioanal. Chem..

[63] Sodowich B.I., Fadl I., Burns C. (2007). Method validation of in vitro RNA transcript analysis on the Agilent 2100 Bioanalyzer. Electrophoresis.

[64] Scorza Francesco B., Yingxia W., Andrew G., Frederick P. (2016). RNA Purification Methods. European Patent.

[65] Wu M.Z., Asahara H., Tzertzinis G., Roy B. (2020). Synthesis of low immunogenicity RNA with high-temperature in vitro transcription. RNA.

[66] Bancel S., Issa W.J., Aunins J.G., Chakraborty T. (2016). Manufacturing Methods for Production of RNA transcripts. U.S. Patent.

[67] Lawrence C. (2020). WP9007: Characterizing Vaccines with Light Scattering. https://patentimages.storage.googleapis.com/7a/bb/8f/5ce58cdaa18a0d/US20160024547A1.pdf.

[68] Hassett K.J., Higgins J., Woods A., Levy B., Xia Y., Hsiao C.J., Acosta E., Almarsson Ö., Moore M.J., Brito L.A. (2021). Impact of lipid nanoparticle size on mRNA vaccine immunogenicity. J. Control. Release.

[69] Chan M.Y., Dowling Q.M., Sivananthan S.J., Kramer R.M. (2017). Particle Sizing of Nanoparticle Adjuvant Formulations by Dynamic Light Scattering (DLS) and Nanoparticle Tracking Analysis (NTA). Methods Mol. Biol..

[70] Packer M., Gyawali D., Yerabolu R., Schariter J., White P. (2021). A novel mechanism for the loss of mRNA activity in lipid nanoparticle delivery systems. Nat. Commun..

[71] Rosa S.S., Prazeres D.M.F., Azevedo A.M., Marques M.P.C. (2021). mRNA vaccines manufacturing: Challenges and bottlenecks. Vaccine.

[72] Kis Z., Tak K., Ibrahim D., Papathanasiou M.M., Chachuat B., Shah N., Kontoravdi C. Pandemic-response adenoviral vector and RNA vaccine manufacturing. medRxiv.

[73] Ibrahim D., Kis Z., Tak K., Papathanasiou M.M., Kontoravdi C., Chachuat B., Shah N. (2021). Model-Based Planning and Delivery of Mass Vaccination Campaigns against Infectious Disease: Application to the COVID-19 Pandemic in the UK. Vaccines.

[74] UNICEF Supply Division (2016). A Process Guide and Toolkit for Strengthening Public Health Supply Chains through Capacity Development.

[75] MOH Kenya Comprehensive Multi-Year Plan for Immunization (2013). July 2015—June Unit of Vaccines and Immunization Services. http://www.nationalplanningcycles.org/sites/default/files/planning_cycle_repository/kenya/kenya_cmyp_2015-2019.pdf.

[76] WHO, PATH (2013). Integration of Vaccine Supply Chains with Other Health Product Supply Chains: A Framework for Decision-Making.

[77] Yadav P., Lydon P., Oswald J., Dicko M., Zaffran M. (2014). Integration of vaccine supply chains with other health commodity supply chains: A framework for decision making. Vaccine.

[78] Chen S.-I., Norman B.A., Rajgopal J., Assi T.M., Lee B.Y., Brown S.T. (2014). A planning model for the WHO-EPI vaccine distribution network in developing countries. IIE Trans..

[79] Lee B.Y., Connor D.L., Wateska A.R., Norman B.A., Rajgopal J., Cakouros B.E., Chen S.-I., Claypool E.G., Haidari L.A., Karir V. (2015). Landscaping the structures of GAVI country vaccine supply chains and testing the effects of radical redesign. Vaccine.

[80] Kis Z., Papathanasiou M., Calvo-Serrano R., Kontoravdi C., Shah N. (2019). A model-based quantification of the impact of new manufacturing technologies on developing country vaccine supply chain performance: A Kenyan case study. J. Adv. Manuf. Process..

[81] Kis Z., Papathanasiou M., Kotidis P., Antonakoudis T., Kontoravdi C., Shah N., Khan M.A., Campa C. (2021). Stability Modeling for Biopharmaceutical Process Intermediates. Quality by Design—An Indispensable Approach to Accelerate Biopharmaceutical Product Development.

[82] Upadhyay S.K. (2007). Chemical Kinetics and Reaction Dynamics.

[83] Chang R. (2005). Physical Chemistry for the Biosciences.

[84] Clénet D., Imbert F., Probeck P., Rahman N., Ausar S.F. (2014). Advanced Kinetic Analysis as a Tool for Formulation Development and Prediction of Vaccine Stability. J. Pharm. Sci..

[85] Peleg M., Normand M.D., Corradini M.G. (2012). The Arrhenius Equation Revisited. Crit. Rev. Food Sci. Nutr..

[86] Arrhenius S. (1889). Über die Dissociationswärme und den Einfluss der Temperatur auf den Dissociationsgrad der Elektrolyte. Zeitschrift Phys. Chemie.

[87] Arrhenius S. (1889). Über die Reaktionsgeschwindigkeit bei der Inversion von Rohrzucker durch Säuren. Zeitschrift Phys. Chemie.

[88] Laidler K.J. (1987). Chemical Kinetics..

[89] Wang W., Singh S., Zeng D.L., King K., Nema S. (2007). Antibody Structure, Instability, and Formulation. J. Pharm. Sci..

[90] Clénet D. (2018). Accurate prediction of vaccine stability under real storage conditions and during temperature excursions. Eur. J. Pharm. Biopharm..

[91] Menzen T., Friess W. (2014). Temperature-ramped studies on the aggregation, unfolding, and interaction of a therapeutic monoclonal antibody. J. Pharm. Sci..

[92] Houde D.J., Berkowitz S.A. (2019). Biophysical Characterization of Proteins in Developing Biopharmaceuticals.

[93] Seeliger D., Schulz P., Litzenburger T., Spitz J., Hoerer S., Blech M., Enenkel B., Studts J.M., Garidel P., Karow A.R. (2015). Boosting antibody developability through rational sequence optimization. MAbs.

[94] Cragnolini T., Derreumaux P., Pasquali S. (2015). Ab initio RNA folding. J. Phys. Condens. Matter.

[95] Ding F., Sharma S., Chalasani P., Demidov V.V., Broude N.E., Dokholyan N. (2008). V Ab initio RNA folding by discrete molecular dynamics: From structure prediction to folding mechanisms. RNA.

[96] Jin L., Shi Y.-Z., Feng C.-J., Tan Y.-L., Tan Z.-J. (2018). Modeling Structure, Stability, and Flexibility of Double-Stranded RNAs in Salt Solutions. Biophys. J..

[97] Rahman M.M., Zhou N., Huang J. (2021). An Overview on the Development of mRNA-Based Vaccines and Their Formulation Strategies for Improved Antigen Expression in Vivo. Vaccines.

[98] Kim S.C., Sekhon S.S., Shin W.-R., Ahn G., Cho B.-K., Ahn J.-Y., Kim Y.-H. (2021). Modifications of mRNA vaccine structural elements for improving mRNA stability and translation efficiency. Mol. Cell. Toxicol..

[99] Schmid A. (2017). Considerations for Producing mRNA Vaccines for Clinical Trials. Methods Mol. Biol..

[100] Uddin M.N., Roni M.A. (2021). Challenges of Storage and Stability of mRNA-Based COVID-19 Vaccines. Vaccines.

[101] Zhang N.-N., Li X.-F., Deng Y.-Q., Zhao H., Huang Y.-J., Yang G., Huang W.-J., Gao P., Zhou C., Zhang R.-R. (2020). A Thermostable mRNA Vaccine against COVID-19. Cell.

